# Sleep-Related Hippocampo-Cortical Interplay during Emotional Memory Recollection

**DOI:** 10.1371/journal.pbio.0050282

**Published:** 2007-10-23

**Authors:** Virginie Sterpenich, Geneviève Albouy, Mélanie Boly, Gilles Vandewalle, Annabelle Darsaud, Evelyne Balteau, Thien Thanh Dang-Vu, Martin Desseilles, Arnaud D'Argembeau, Steffen Gais, Géraldine Rauchs, Manuel Schabus, Christian Degueldre, André Luxen, Fabienne Collette, Pierre Maquet

**Affiliations:** 1 Cyclotron Research Centre, University of Liège, Liège, Belgium; 2 Department of Neurology, Centre Hospitalier Universitaire de Liège, Domaine Universitaire du Sart Tilman, Liège, Belgium; 3 Department of Psychiatry, Centre Hospitalier Universitaire de Liège, Domaine Universitaire du Sart Tilman, Liège, Belgium; 4 Department of Cognitive Sciences, University of Liège, Liège, Belgium; 5 Department of Psychology, University of Salzburg, Salzburg, Austria; Stanford University, United States of America

## Abstract

Emotional events are usually better remembered than neutral ones. This effect is mediated in part by a modulation of the hippocampus by the amygdala. Sleep plays a role in the consolidation of declarative memory. We examined the impact of sleep and lack of sleep on the consolidation of emotional (negative and positive) memories at the macroscopic systems level. Using functional MRI (fMRI), we compared the neural correlates of successful recollection by humans of emotional and neutral stimuli, 72 h after encoding, with or without total sleep deprivation during the first post-encoding night. In contrast to recollection of neutral and positive stimuli, which was deteriorated by sleep deprivation, similar recollection levels were achieved for negative stimuli in both groups. Successful recollection of emotional stimuli elicited larger responses in the hippocampus and various cortical areas, including the medial prefrontal cortex, in the sleep group than in the sleep deprived group. This effect was consistent across subjects for negative items but depended linearly on individual memory performance for positive items. In addition, the hippocampus and medial prefrontal cortex were functionally more connected during recollection of either negative or positive than neutral items, and more so in sleeping than in sleep-deprived subjects. In the sleep-deprived group, recollection of negative items elicited larger responses in the amygdala and an occipital area than in the sleep group. In contrast, no such difference in brain responses between groups was associated with recollection of positive stimuli. The results suggest that the emotional significance of memories influences their sleep-dependent systems-level consolidation. The recruitment of hippocampo-neocortical networks during recollection is enhanced after sleep and is hindered by sleep deprivation. After sleep deprivation, recollection of negative, potentially dangerous, memories recruits an alternate amygdalo-cortical network, which would keep track of emotional information despite sleep deprivation.

## Author Summary

Declarative memories, which can be consciously and verbally retrieved, are initially critically dependent on the hippocampus. However, reliable retrieval of long-term memory depends on a process of consolidation, which partly occurs during sleep, when memories are thought to be progressively transferred to long-term cortical stores. Because people tend to remember emotional memories better than neutral ones, we wondered whether the emotional significance of a memory would enhance its consolidation in a sleep-dependent manner. During a first session, participants viewed pictures with neutral and emotional content without realizing that their memory of the pictures and their content would be tested later (called incidental encoding). Three days later, during a functional MRI scanning session, subjects indicated whether they recognized previously viewed and new pictures. Half of the subjects were totally sleep deprived during the first post-encoding night, but all subjects slept as usual during the second and third post-encoding nights. We show here that the recollection of emotional stimuli elicited larger responses in the hippocampus and various cortical areas in the well-rested group than in the sleep-deprived group, suggesting that emotional significance boosts memory consolidation of the information during sleep. Interestingly, in sleep-deprived subjects, recollection of negative items recruited another network including the amygdala, as if an alternate consolidation process allowed them to keep track of negative, potentially dangerous, information despite the cognitive aftermath of sleep deprivation.

## Abstract 3

A new fMRI study reveals that emotional memories are consolidated by different brain networks in humans who receive a normal amount of sleep, compared with those who are sleep deprived.

## Introduction

Memory consolidation is the protracted process by which fresh, initially labile, memories are reorganized into enduring stable memories [1]. At the systems level, memory consolidation results in a progressive rearrangement of memories that can eventually be stored in circuits different from those in which they were initially encoded [2]. For instance, declarative memories, which are originally heavily dependent on mesio-temporal structures, are thought to be gradually restructured in mature memories, stored in more distributed cortical networks [2,3], although not necessarily exclusively therein [4].

A growing body of data suggests that sleep is involved in the consolidation of declarative memories [5]. At the behavioral level, retention of the encoded information, when tested at a later date, is often significantly higher if sleep follows encoding within a few hours [6]. Sleep also protects declarative memories from subsequent interference [7]. Moreover, training to a declarative memory task is followed by changes in fine-grained sleep structure during the post-encoding night, such as an increase in sleep spindle density [8]. In contrast, sleep deprivation hinders the plastic changes that normally occur during sleep and alters the brain responses subsequently recorded at retest [9].

Although still elusive and a matter of intense research, the basic mechanisms underpinning memory consolidation during sleep include both cellular (e.g., synaptic homeostasis [10]) and systemic processes. At the systems level, they seem to consist in the replay of firing sequences in neuronal populations involved in learning. Evidence for a replay of neuronal firing sequences has been collected in rodents during both slow-wave sleep [11,12] and rapid eye movement (REM) sleep[13] within the hippocampus, as well as in the neocortex [14,15]. In addition, during non-REM sleep, a significant correlation has been reported between hippocampal and neocortical neuronal discharges, respectively structured by sharp waves/ripples, and sleep spindles [16,17] or slow waves [18]. Consistent with data collected in rodents, task-related, experience-dependent increases in the hippocampal and neocortical activity were detected in humans during non-REM sleep following spatial learning [19].

Emotional events are usually better remembered than neutral ones are [20]. This bias toward a better recollection of emotional memories is mediated by the amygdala, which modulates the activity in the hippocampus and thereby influences the consolidation of emotional memories [21]. In humans, functional relationships suggestive of a modulation of the hippocampus by the amygdala were detected not only during encoding [22] or retrieval [23], but also during the consolidation period [24]. Based on the evidence presented above, it can be sensibly assumed that the consolidation of emotional memories would also benefit from sleep. In line with this hypothesis, aversive as well as appetitive conditioning in rodents is followed by an increase in REM sleep [25]. The conditioned response, learned during wakefulness, can be elicited in the amygdala during REM sleep following aversive conditioning [26]. Conversely, sleep deprivation impairs contextual fear conditioning [27]. The hypothesis of a role of sleep in the consolidation of emotional memories has not been often addressed in human literature. Yet, the amygdala and the hippocampus are among the most active brain structures during REM sleep [28], a condition which is likely to favor amygdalo-hippocampal interactions. An early report observed an impaired recall of threatening material if participants were deprived of REM sleep [29]. Consistent with this finding, the retention of emotional texts is enhanced if sleep was allowed in the second part of the night, when REM sleep predominates [30]. This effect, which involves a modulation by glucocorticoids [31] can still be observed 4 y after encoding [32]. Likewise, there is a selective memory enhancement for arousing picture after 12 h of sleep as compared with 12 h of wakefulness [33].

**Figure 1:**
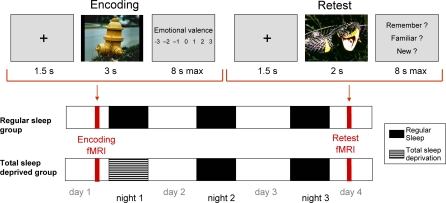
Experimental Protocol

In the present study, we examined the impact of sleep and total sleep deprivation on the consolidation of emotional memories in humans using functional neuroimaging. During a first functional MRI (fMRI) scanning session, neutral and emotional pictures were incidentally encoded by all participants (Figure 1). Half of the subjects were totally sleep deprived during the first post-encoding night. These two groups of subjects are referred below to as sleeping (resting sleep, RS) and sleep-deprived (total sleep deprivation, TSD) groups, although in all cases, the subjects slept as usual during the second and third post-encoding nights. Three days later, during a second fMRI scanning session, subjects made recognition memory judgments about previously studied pictures and new pictures. For each stimulus, the subjects indicated whether they could retrieve specific details about the encoding episode (“Remember” responses), if they just had a feeling of familiarity (“Know” responses) [34], or if they thought the item had not been presented during encoding (“New” responses).

We hypothesized that: (1) emotional events would be better remembered than neutral ones; (2) sleep and sleep deprivation during the post-encoding night would differentially influence subsequent explicit recognition of emotional and neutral items; and (3) sleep deprivation would disrupt the slow processes that underpin memory consolidation during sleep, thereby modifying the neural correlates of successful recognition of emotional (as compared to neutral) items during subsequent testing.

## Results

### Sleep Parameters

Sleep duration and quality were assessed by questionnaires during the experiment (Table S1 and Text S1). Mean subjective sleep duration was not significantly different between groups on the nights preceding the encoding and the retest session (night before encoding: *F*(1,37) = 0.45, *p* = 0.5; night before retest: *F*(1,37) = 1.14, *p* = 0.3). Likewise, mean subjective sleep quality (rating on a ten point scale) was equivalent between groups for these nights (night before encoding: *F*(1,37) = 0.006, *p* = 0.94; night before retest: *F*(1,37) = 0.01, *p* = 0.9). Mean subjective sleep duration was longer for the TSD than for the RS group for the second post-encoding night (*F*(1,37) = 17.45, *p* < 0.0001), reflecting the expected sleep rebound after deprivation. Subjective sleep quality appears to be unaffected on this night (*F*(1,37) = 1.56, *p* = 0.22).

Actigraphic data recorded during four nights (one before, three after encoding) differed between groups (*F*(1,31) = 115.08, *p* < 0.001) and between nights (*F*(3,93) = 168.1, *p* < 0.001). The group by night interaction was also significant (*F*(3,93) = 157.9, *p* < 0.001). There was no significant difference between groups in the activity during the night before encoding (*F*(1,31) = 0.13, *p* = 0.71). As expected, the activity was larger in the TSD than in the RS group during the first night (*F*(1,31) = 159.4, *p* < 0.001). During the second night, activity in TSD subjects was lower than in RS subjects, suggesting a rebound of deep sleep after sleep deprivation (*F*(1,31) = 4,19, *p* = 0.049). This effect was no longer present on the third night, which preceded the retest (*F*(1,31) = 1.93, *p* = 0.17), suggesting that two nights are sufficient to recover from sleep deprivation.

### Behavioral Results

An analysis of variance (ANOVA) on memory performance with memory type (Remember [R] versus Know [K]) and emotion (Negative [Neg] versus Neutral [Neu] versus Positive [Pos]) as within-subjects factors and sleep group (RS versus TSD) as between-subjects factor showed a significant main effect of sleep on all hits (R + K responses, *F*(1,37) = 5.2, *p* = 0.03). This result indicates that sleep deprivation on the post-encoding night alters global memory consolidation (Table 1).

**Table 1:**
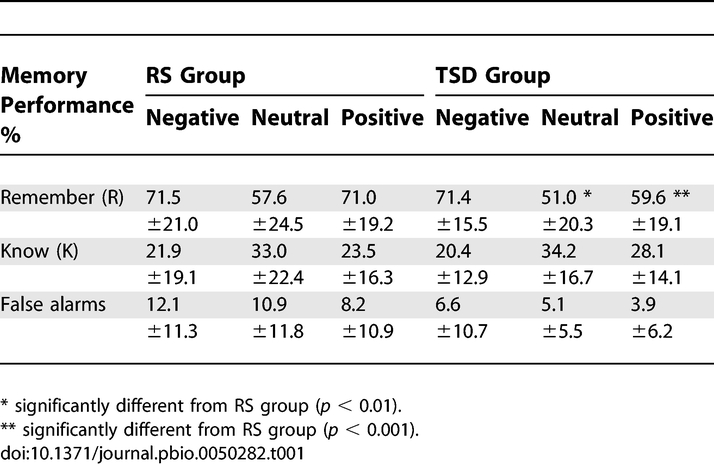
Behavioral Data (mean ± SD)

We also observed a significant main effect of emotion (*F*(2,74) = 7.5, *p* = 0.001). The emotion by sleep interaction showed only a trend towards significance (*F*(2,74) = 2.4374, *p* = 0.094). More interestingly, sleep effect was significant on hits for neutral (*F*(1,37) = 4.41; *p* = 0.04) and positive stimuli (*F*(1,37) = 7.31; *p* = 0.01), but not for negative items (*F*(1,37) = 0.49; *p* = 0.49).

**Figure 2:**
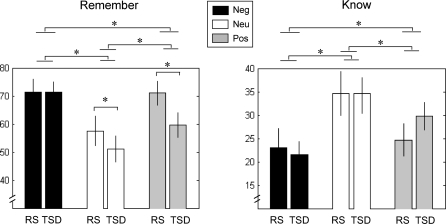
Behavioral Data Percentage of correctly remembered and known images as a function of sleep groups. RS: regular sleep, TSD: total sleep deprivation, Neg: negative, Neu: neutral, Pos: Positive.

There was a main effect of memory type (R versus K responses; *F*(1,37) = 48.68, *p* < 0.001). The emotion by memory type interaction was significant (*F*(2,74) = 20.33, *p* < 0.001, Figure 2). Both negative and positive pictures were better remembered than neutral ones (planned comparisons, Neg-R > Neu-R: *p* < 0.001, Pos-R > Neu-R: *p* < 0.001), and negative pictures were significantly better remembered than positive ones (*p* = 0.02). Moreover, negative and positive pictures induced less K responses than neutral ones (Neg-K < Neu-K: *p* < 0.001, Pos-K < Neu-K: *p* = 0.002), whereas negative pictures induced more K responses than positive ones (Neg-K > Pos-K: *p* = 0.04, Table 1).

The triple interaction between emotion, memory, and sleep was not significant (*F*(2,74) = 1.7, *p* = 0.18): emotional events were not differently recognized than neutral ones after sleep or sleep deprivation (Figure 2). However, recollection of neutral items deteriorated after sleep deprivation (51.0% versus 57.6% in the RS group, 7.6% decrease, *p* < 0.01). Recollection of positive items was even more altered by sleep deprivation (59.6% versus 71.0% in the RS group, 11.4% decrease, *p* < 0.001). In contrast, performance was unaffected by lack of sleep for negative stimuli (71.4% versus 71.5%, *p* = 0.97, post-hoc LSD Fisher tests). We observed no significant difference in the number of K responses for the three emotional valences according to sleep (RS versus TSD, Neg-K: *p* = 0.77, Neu-K: *p* = 0.86, Pos-K: *p* = 0.35).

An ANOVA computed on false alarms (new pictures identified as old) shows a tendency for the effect of sleep (*F*(1,36) = 3.5, *p* = 0.07), but shows neither a significant effect of emotion (*F*(2,72) = 2.19, *p* = 0.12) nor a significant emotion by sleep interaction (*F*(2,72) = 0.16, *p* = 0.85). These results do not support the hypothesis of a general response bias due to emotion but leave an effect of sleep uncertain.

To further assess the possibility of a response bias due to sleep, we performed an analysis on the discrimination index (d') and on the criterion according the procedure of Snodgrass and Corwin [35]. No effect of sleep was detected on the discrimination index (d', mean ± standard deviation [SD]: RS group = 3.06 ± 0.86, TSD group = 3.21 ± 0.81, *t*(1,37) = −0.56, *p* = 0.58), but the criterion significantly differed between groups (*t*(1,37) = −2.31, *p* = 0.026). The mean criterion was close to zero in the sleep group (mean ± SD : −0.005 ± 0.54), suggesting that these subjects were unbiased in their decisions. In contrast, the mean criterion in the sleep deprived group was 0.38 ± 0.50, suggesting that these subjects were slightly more conservative.

The pupillary size, taken as an index of autonomic response to a given stimulus, was monitored during both the encoding and retest sessions (Table 2). We computed an ANOVA with emotion (Neg, Neu, Pos) and session (encoding and retest) as within-subjects factors. There was a significant effect of session (*F*(1,37) = 8.84, *p* = 0.005), the pupil being larger during encoding than retest, suggesting a larger arousal on the first presentation of the stimuli. As expected [36], pupillary data showed a significant main effect of emotion (*F*(2,74) = 42.16, *p* < 0.001). We observed a significant interaction between emotion and session (*F*(2,74) = 6,718, *p* = 0.002). During encoding and retest, planned comparison showed that pupillary size was larger after presentation of negative than neutral pictures (encoding: *F*(1,37) = 97.3, *p* < 0.001, retest: *F*(1,37) = 25.31, *p* < 0.001), suggesting that negative pictures induced a larger autonomic response than neutral ones. In contrast, positive pictures did not elicit a change in pupillary size compared to neutral ones during encoding (*F*(1,37) = 0.12, *p* = 0.73). During retest, pupillary size even decreased after the presentation of positive pictures as compared to neutral ones (*F*(1,37) = 7.50, *p* = 0.009).

**Table 2:**
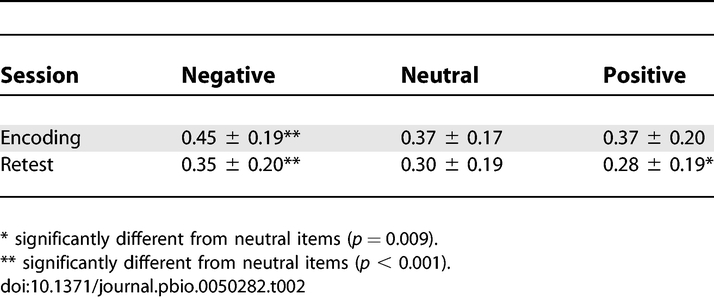
Pupillary Data [Difference in Pupillary Size between the Presentation of the Picture and the Baseline (mm)]

### Functional MRI Results

For the sake of completeness, several main effects are reported in the supplemental material: (1) main effect of successful recognition (R + K responses) separately for the three valences in both groups (Table S3, Figure S1A), (2) the effects of emotion on successful recognition in both groups (negative versus neutral, positive versus neutral, negative versus positive, Table S4, Figure S1B), and (3) the main effect of memory type in both groups (R versus K responses, Table S5, Figure S2). From these data, one result is worth mentioning. Successful recognition of negative items recruited a larger thalamo-hippocampo-cortical network, which included the amygdala, than either neutral or positive items recruited. In contrast, a significant difference in brain response during successful recognition of positive relative to neutral items was only detected in the medial prefrontal cortex.

Our predictions concern the effects of sleep on explicit memory consolidation, and so we concentrate below on the contrast between recollection (R responses) and familiarity (K responses), because it probes the memory processes that precisely contribute to explicit retrieval.

First, we observed that responses elicited by successful recollection (R > K) of stimuli, irrespective of their emotional valence, were significantly larger in RS than in TSD group in the medial prefrontal cortex and in the hippocampus (memory by sleep interaction, Table 3) .

**Table 3:**
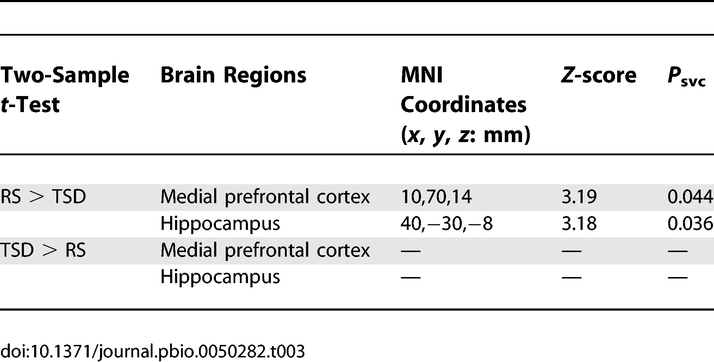
Effect of Memory (R > K) (Neg + Neu + Pos) during Retest

We next examined whether there was any difference in brain responses for remembered relative to known items, depending on their emotional content (emotion by memory interaction). First, we present results for the contrast for negative versus neutral items [(R > K) × (Neg > Neu)]. For subjects allowed to sleep on the post-encoding night, a significant interaction was observed in a set of neocortical areas, including the medial prefrontal cortex, the superior frontal gyrus, the superior temporal sulcus, the occipital cortex, and in the anterior cingulate cortex (Table 4). In contrast, in the amygdala, posterior probability of activation, as inferred by Bayesian statistics, was low (16%). For sleep-deprived subjects, a significant memory by emotion interaction was detected in the amygdala and the superior temporal sulcus (Table 4), whereas posterior probability of activation in the hippocampus was negligible (7%).

**Table 4:**
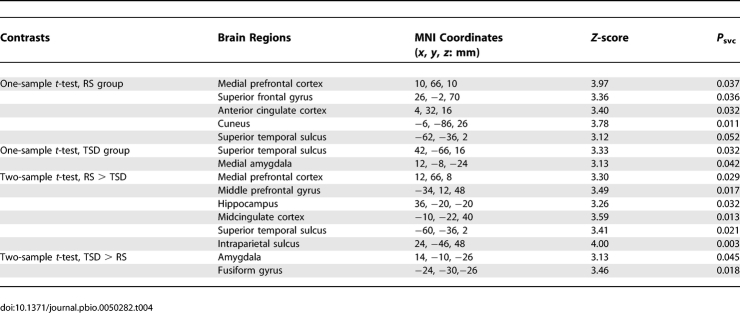
Interaction between Emotion and Memory (Neg > Neu) × (R > K) during the Retest

**Figure 3:**
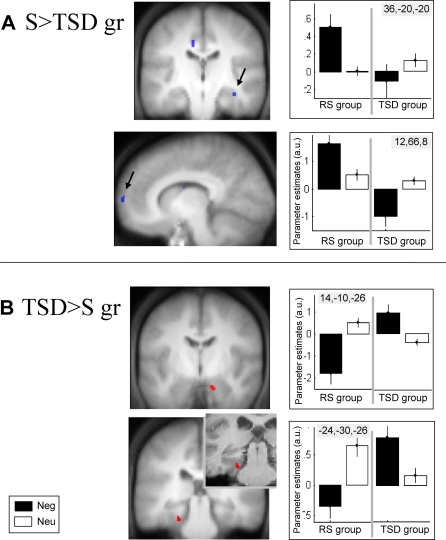
Effect of Sleep on Emotional (Negative) Memory (A) Memory (R > K) × emotion (Neg > Neu) × sleep status (RS > TSD) interaction. From the top to the bottom: the hippocampus and the medial prefrontal cortex. (B) Memory (R > K) × emotion (Neg > Neu) × sleep status (TSD > RS) interaction. From the top to the bottom: amygdala and fusiform gyrus (inset: enlarged mesio-temporal region in a representative subject). Neu: neutral; Neg: negative. Left panels: functional results are displayed on the mean structural MR image, normalized to the same stereotactic space (display at *p* < 0.001, uncorrected). Right panels: parameter estimates of recollection minus familiarity (arbitrary units ± SEM).

The memory by emotion interaction significantly differed between groups. As compared with the TSD group, the RS group was characterized by larger responses in the right hippocampus, the medial prefrontal cortex, the middle prefrontal gyrus, the mid-cingulate cortex, the superior temporal sulcus, and the intraparietal sulcus (Table 4, Figure 3A). As shown by the parameter estimates, in the RS group, the response in these regions was larger for negative than neutral pictures, and more so for the remembered than known items, whereas the reverse pattern was observed in the TSD group. Conversely, the responses were significantly larger in the TSD than in the RS group in two regions, the amygdala and the antero-medial part of the fusiform gyrus (transverse collateral sulcus abutting to parahippocampal gyrus). These two regions respond more to negative than to neutral pictures, in the context of recollection (as compared to familiarity) in the TSD group than in the RS group (Table 4, Figure 3B).

The interactions concerning negative relative to positive items essentially yielded similar results, although no significant response was identified in the hippocampus and amygdala (see Table S6). However, masking inclusively the Neg > Neu contrast by the Neg > Pos contrast (threshold, *p* < 0.05) confirmed that responses in these two structures were common to both contrasts, although at different statistical threshold.

**Figure 4:**
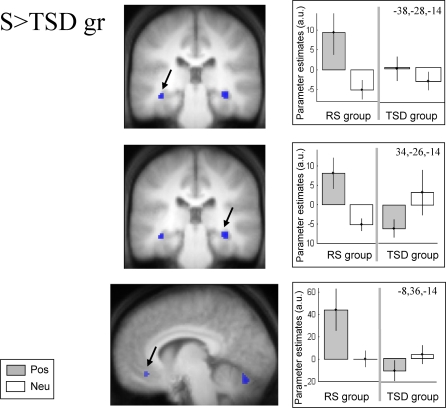
Effect of Sleep on Emotional (Positive) Memory Modulated by Performance (A) Memory (R > K) × emotion (Pos > Neu) × sleep status (RS > TSD) interaction. From the top to the bottom: both hippocampi and the medial prefrontal cortex. Pos: positive; Neu: neutral. Left panels: functional results are displayed on the mean structural MR image, normalized to the same stereotactic space (display at *p* < 0.001, uncorrected). Right panels: parameter estimates of recollection minus familiarity (arbitrary units ± SEM).

We also characterized the interaction between emotion and memory for positive versus neutral items [(R > K) × (Pos > Neu)]. Significant differential responses were detected in a few scattered areas, which have been previously reported in memory literature but are not considered as crucial nodes for declarative retrieval (Table 5). This paucity of results was puzzling given that, behaviorally, sleep deprivation significantly deteriorated the recollection of positive items, suggesting a difference in brain responses between RS and TSD. However, it should be kept in mind that the reported interactions only concerned correct responses and thus were independent of the overall recollection rate. To take into account the effect of sleep deprivation on recollection, we conducted another multiple regression analysis at the level of random effects, looking at brain areas that respond more to recollection of positive than neutral items in proportion to the overall individual memory performance (proportion of hits). This multiple regression analysis corresponds to a memory by emotion by performance interaction and characterizes the brain network that is recruited to a larger extent in good than bad performers, and more so for positive than neutral recollected items [(R > K) × (Pos > Neu) × performance]. The subjects who were allowed to sleep showed significantly larger responses during recollection of positive than neutral items in both hippocampi and in the medial prefrontal cortex, superior frontal gyrus, and superior temporal sulcus, in proportion to the overall individual memory performance (Table 6). The responses in the hippocampus, medial prefrontal cortex, and insula were significantly more tightly related to memory performance in sleep than sleep deprived subjects (Table 6 and Figure 4). As shown by the parameter estimates, in proportion to individual memory performance, the response in these regions was larger for positive than neutral recollected items, and more so in the RS than the TSD group. In contrast, no significant results were observed for sleep deprived subjects.

**Table 5:**
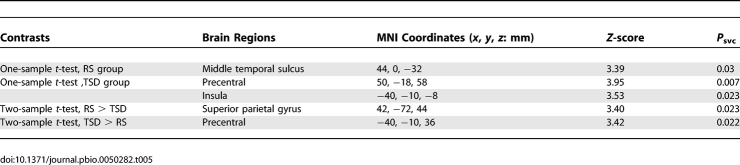
Interaction between Emotion and Memory (Pos > Neu) × (R > K) during Retest

**Table 6:**
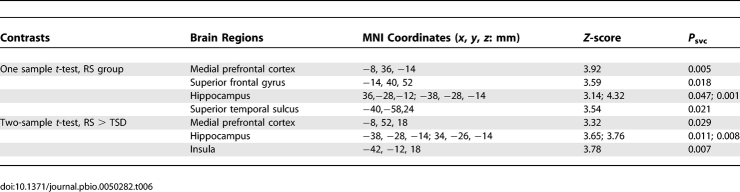
Interaction between Emotion and Memory (R>K) x (Pos>Neu) Modulated by Performance during Retest

For the sake of symmetry, the same analysis was conducted for negative versus neutral stimuli [(R > K) × (Neg > Neu) × performance]. In proportion to their individual memory performance, subjects allowed to sleep showed significantly larger responses for recollection of negative than neutral items in the right hippocampus and medial prefrontal cortex as well as in the precuneus, the anterior and retrosplenial cingulate cortices, and the middle temporal cortex (Table 7). The response in the hippocampus, the retrosplenial cingulate cortex and precuneus was significantly larger in sleep than in sleep-deprived subjects. In contrast, sleep-deprived subjects significantly recruited the amygdala for negative as compared to neutral items during successful recollection, in proportion to individual performance. This response was significantly larger in sleep-deprived than in sleeping subjects (Table 7).

**Table 7:**
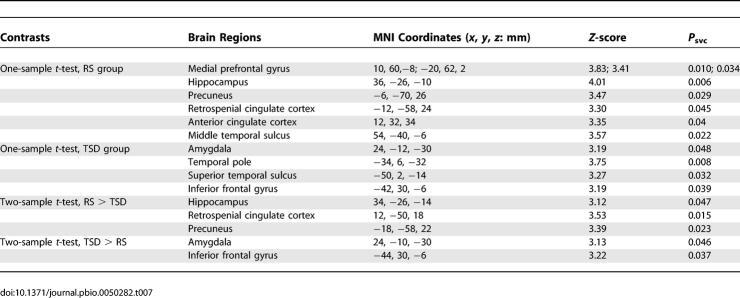
Interaction between Emotion and Memory (Neg > Neu) × (R > K) Modulated by Performance during Retest

**Figure 5:**
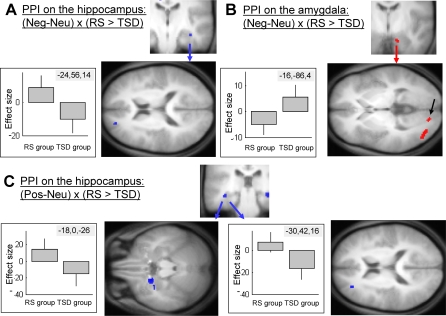
Psychopysiological Interactions (A and B) Regions more connected to seed areas [(A) right hippocampus, (B) amygdala] for negative than neutral correctly remembered pictures (inset: enlarged prefrontal region in a representative subject). (C) Regions are more connected to the left hippocampus for positive than neutral correctly remembered pictures.

We also tested whether the functional connectivity of two reference regions would establish distinct functional connections with other brain regions, in the context of emotional as compared to neutral hits, and depending on whether subjects were allowed to sleep during the post-encoding night. The seed regions were the right hippocampus and the amygdala, identified in the emotion (Neg > Neu) × memory × sleep interaction described above as well as the left hippocampus, identified in the emotion (Pos > Neu) × memory × sleep interaction. The right hippocampus was more connected to the superior frontal sulcus, anterior parts of superior temporal sulcus, inferior temporal gyrus, and the thalamus, in RS than in the TSD group and more for negative than neutral hits (Table 8, Figure 5A). The left hippocampus was more connected to the middle frontal gyrus, the right hippocampus, and the amygdala, in the RS than in the TSD group and more for positive than neutral hits (Table 8, Figure 5C). As shown by the parameter estimates, these regions are more connected to the hippocampus for the RS than for the TSD group. On the other hand, the amygdala was found functionally more tightly connected to the orbitofrontal cortex, striate and extrastriate cortices, the superior temporal gyrus, and the posterior insula, in the TSD than the RS group and more so for negative than neutral hits (Table 8, Figure 5B). As shown by parameter estimates, these regions are more connected to the amygdala in the TSD than in the RS group.

**Table 8:**
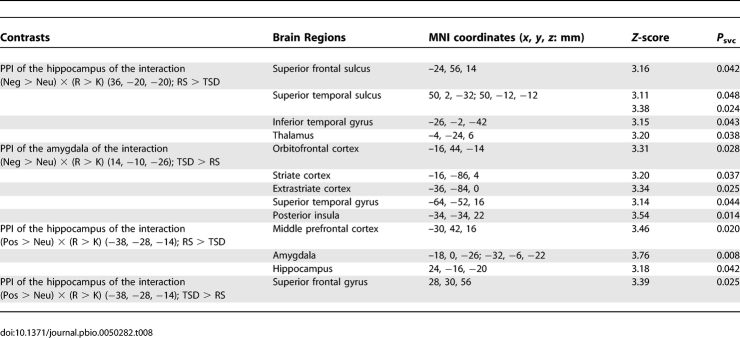
PPI on Seed Areas Taken from the Interactions (Neg > Neu) × (R > K) (Right Hippocampus and Amygdala) and (Pos > Neu) × (R > K) (Left Hippocampus) during Retest

Finally, to check that the responses observed during testing were similar to initial responses to emotional stimuli, we assessed the effect of emotion (Neg > Neu) during encoding. Significant responses were observed in similar areas in both groups, including the amygdala, the fusiform gyrus, the middle occipital gyrus, the prefrontal cortex, the middle and posterior cingulate cortices, the insula, and the anterior temporal cortex. A large right precentral area was also activated, because subjects had to press on a button with their left hand to rate the picture valence. We did not find any significant difference in the effect of emotion between groups, confirming that both groups processed emotions in a similar way during encoding (Table 9).

**Table 9:**
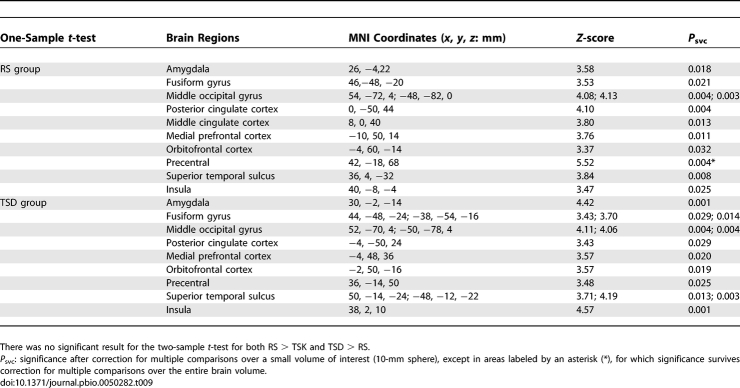
Effect of Emotion (Neg > Neu) during Encoding

## Discussion

The neural correlates of successful recollection of emotional and neutral stimuli were assessed using fMRI, 72 h after encoding, with or without an intervening total sleep deprivation during the first post-encoding night. As expected, total sleep deprivation had a significant negative impact on global memory performance, in keeping with previous reports on the deleterious effects of sleep deprivation on declarative memory [37]. Nevertheless, no significant difference in recollection was observed between groups. Intriguingly, this lack of effect was related to a strong influence of stimulus valence on recollection rates: recollection of negative stimuli was not affected by lack of sleep, whereas recollection of neutral and even more-so positive items was significantly deteriorated after sleep deprivation.

The main finding of the present study is that recollection of emotional (as compared to neutral) items is associated with increased responses in the hippocampus and various cortical areas, including the medial prefrontal cortex, in subjects allowed to sleep, relative to sleep-deprived participants. This finding suggests that the emotional meaning of the learned material enhances the consolidation of declarative memories during sleep.

A second important finding is that the recollection of negative items after sleep consistently elicits the hippocampo-neocortical response pattern in all subjects. In contrast, for positive items, the recruitment of this response pattern varies across subjects, in proportion to their individual memory performance. This finding suggests that the superiority of negatively-valenced material in systematically enhancing sleep-dependent memory offline processing.

A third finding is that successful recollection of negative items in sleep-deprived subjects elicits larger responses in an amygdalo-cortical network than in the sleep group. No such difference between groups is observed for positive stimuli. We suggest that this alternate pathway allow sleep-deprived participants to achieve the same recollection level for negative items than subjects allowed to sleep.

### Sleep-Dependent Changes in Hippocampal and Neocortical Responses Elicited by Successful Recognition

In this experiment, the effects of sleep on memory consolidation were probed indirectly, by characterizing the differences in brain responses elicited during retrieval, depending on whether the participants were allowed to sleep on the first post-encoding night. In the absence of an immediate recognition test, we could not assess whether responses induced by recollection changed over the first 72 h post-encoding in both groups, or, in other words, if some aspects of memory consolidation went on irrespective of whether participants slept on the first post-encoding night. In contrast, our results show that sleeping on this particular night has an effect on brain responses subsequently recorded during recognition. Larger responses were observed in subjects who were allowed to sleep than in sleep-deprived subjects, in the medial prefrontal cortex as well as in the hippocampus, irrespective of the emotional valence of the learned stimuli (Table 3). These findings suggest that sleep deprivation affects hippocampal activity not only if it precedes encoding [47] but also if it takes place during the consolidation period. These changes in cortical responses support the view of a progressive engagement of cortical circuits during the course of memory consolidation. In animals, the reorganization of brain responses underlying systems-level consolidation of hippocampus-dependent memories develops over weeks [38–40]. However, behavioral impairment can be detected within hours or days after training when molecular processes of memory consolidation are hindered [41,42]. In humans, the time course of systems-level memory consolidation is not well known. Retrograde amnesia in patients with bilateral hippocampal lesions usually spans several years [43]. However, in normal subjects, ventral medial prefrontal responses increase nonlinearly within weeks after encoding [44]. Our data extend this finding, in showing that the progressive recruitment of medial prefrontal cortex in the course of memory consolidation starts early on, during the first post-training night, and is promoted by offline processes taking place during sleep.

The medial prefrontal area reported here is anterior and rostral to the coordinates previously reported [44]. This anterior location might be related to the memory task. Responses specific to explicit recollection were reported in various portions of the medial prefrontal cortex including fronto-polar regions [45]. Alternatively, based on our experimental design, it can be suggested that the anterior medial prefrontal response reflects an early consolidation process, possibly related to sleep. Early on during consolidation, memory retrieval might activate a large part of medial-frontal cortex and involve a number of highly interconnected [46] medial frontal areas. As consolidation progresses, the activation would gradually converge to the pregenual medial frontal region[44].

### Influence of Emotion on Sleep-Dependent Changes in Hippocampal and Neocortical Responses

Recollection of emotional (as compared to neutral) items is associated with increased responses in the hippocampus and various cortical areas, including medial prefrontal cortex, in subjects allowed to sleep relative to sleep-deprived participants. Moreover, in sleeping subjects, functional connectivity between the hippocampus and the medial prefrontal cortex (among other cortical areas) was tighter than in sleep-deprived subjects during the recollection of emotional items (as compared to neutral). These findings suggest that the emotional significance of the learned material enhances the consolidation of declarative memories during sleep.

We did not record the offline processes, which took place during the first post-encoding night at both cellular and systems levels, and which lead to brain responses observed during recollection. However, we assumed that the amygdala modulates hippocampal activity during sleep in such a way that the hippocampal-neocortical dialogue is enhanced. Intriguingly, consolidation of declarative memories is often related to non-REM sleep, whereas the emotion-related modulation by the amygdala of the hippocampus is presumed to occur during REM sleep [29,30]. Future research should specify the respective role of REM and non-REM sleep (or their ordered succession throughout the night) in emotional memory consolidation.

In the sleep group, the absence of amygdalar response during recollection of emotional memories is not necessarily surprising. Some authors report an amygdalar response during retrieval [23], even at long retention delay [48], whereas others do not [24,49]. Our results indicate that if consolidation progresses without sleep deprivation, a hippocampal-cortical network can keep track of the emotional meaning of the encoded items, at least during the first days of the consolidation period.

### Differential Influence of Negative and Positive Valence on Sleep-Dependent Changes in Hippocampal and Neocortical Responses

Successful recollection of negative items after sleep (relative to those after sleep deprivation) consistently elicited a hippocampo-neocortical response pattern across all subjects. In contrast, recollection of positive items was not associated with different brain responses between groups, except in the superior parietal cortex. This paucity of results was intriguing, because in contrast to negative stimuli, recollection of positive items is deteriorated by sleep deprivation. However, the reported interaction only characterized brain responses associated with hits and was independent on the overall memory performance of each participant. To account for behavioral performance levels in the analysis of fMRI data, we conducted another analysis, which included the individual hit rates. Consistent with our hypothesis, responses associated with successful recollection were larger in the hippocampus and medial prefrontal cortex for positive than neutral items, and more so in the RS than in the TSD group, but only in proportion of individual memory performance. This contrast indicates that the observed changes in brain responses are influenced by two main factors: the positive valence of the recollected events and sleep during the first post-training night. Interindividual differences in retrieval processes (retrieval effort, source and response monitoring, decision bias, etc.) are unlikely to explain this result, because they would equally apply to neutral and positive items. One might also argue that the variability in recollection rates arises from differences in encoding between emotional and neutral items. However, this explanation would not account for the difference in brain responses observed during recollection between RS and TSD groups. In consequence, this result points to an effect of sleep deprivation disrupting to a larger extent the consolidation of positive than neutral items.

Collectively, these findings suggest that negative emotional memories trigger sleep-dependent consolidation processes more consistently than positive items do. Because positive and negative stimuli elicit different levels of arousal both during encoding and retrieval, as confirmed by the pupillary size (Table 2), the arousing dimension of an emotional stimulus might also influence its subsequent consolidation. For instance, specific differences in neuromodulation underlying the processing of positive [50] and negative [51] stimuli might modify their processing offline.

### An Alternate Response Pattern for Recollection of Negative Stimuli after Sleep Deprivation

In sleep-deprived subjects (as compared with sleeping subjects), recollection of negative (as compared with neutral) items involved the fusiform gyrus and the amygdala, the latter being preferentially functionally connected to the orbito-frontal cortex and several posterior (occipito-temporal) areas. We hypothesized that this restricted cortical circuit reflects an alternate consolidation process, which developed despite, or owing to, sleep deprivation. The resulting network is reminiscent of the response recorded during the initial exposure to emotional material ([52], see the effect of emotion during encoding), indicating that the memory has been processed only to a limited extent. This finding also suggests that the amygdala can partially store emotional memories when consolidation has been hindered by sleep deprivation. Likewise, the significant responses observed in the fusiform gyrus can be interpreted as a storage site for the encoded visual information [53]. Alternatively, recollection of emotional items would emerge from the coordinated recruitment of the orbito-frontal cortex, the occipital cortex, and the amygdala. This network possibly interacts with the hippocampal-neocortical circuits, which we otherwise observe in response to recollection of items irrespective of their emotional valence (see Table S5).

We could not identify a similar alternate response for positive items after sleep deprivation. We hypothesize that after sleep deprivation, the recruitment of the alternate amygdalo-cortical network for negative items supports memory retrieval and explains that recollection levels are similar to those achieved after sleep. In the absence of such alternate pathway for positive items, their recollection rate is decreased after sleep deprivation

### Conclusions

Collectively, our data are consistent with an early engagement of neocortical areas, especially of medial frontal areas during memory consolidation. The recruitment of hippocampo-neocortical networks during recollection is enhanced after sleep and is hindered by sleep deprivation, indicating that sleep promotes systems-level memory consolidation. The emotional significance of the encoded information further enhances these sleep-dependent processes. However, after sleep deprivation, an alternate amygdalo-neocortical network is recruited during recollection of negative but not positive stimuli. Recollection of negative, emotionally arousing stimuli seems to rely on redundant neural systems. This degeneracy offers an adaptive advantage that allows us to keep track of emotionally arousing, potentially dangerous environmental features despite the cognitive aftermaths of sleep deprivation.

## Materials and Methods

### Subjects.

Forty normally-sighted healthy volunteers (23 women, 17 men, mean age 22.3 ± 2.7 years) gave their written informed consent to take part in this fMRI study, which was approved by the Ethics Committee of the Faculty of Medicine of the University of Liège. None had any history of trauma or of medical, psychiatric, or sleep disorder (Text S1). They followed a 7-d constant sleep schedule (according to their own rhythm ± 1 h) before the first visit and kept the same schedule for 3 more days, until their second visit. Compliance to the schedule was assessed, using both sleep diaries and wrist actigraphy (Actiwatch, Cambridge Neuroscience).

### Experimental material.

Intrinsically emotional stimuli were used to guarantee a sufficient recognition rate after 3 d. The set of stimuli was taken from the International Affective Pictorial System [54]. They consisted of 160 emotional pictures (80 unpleasant, mean valence on a 9-point scale: 2.87 ± 0.66; 80 pleasant, mean valence: 7.4 ± 0.48; and 80 neutral, mean valence: 5.4 ± 0.65) (Table S2). Each of the valence categories contained a similar proportion of objects, landscapes, animals, and humans. The luminance of the pictures was checked and equalized.

### Experimental protocol.

Each subject was scanned during two separate sessions (Figure 1). During the first session (encoding), 40 pictures of each valence were presented in random order. Each picture was displayed for 3 s (17° × 23°). After the stimulus disappeared, the subjects had a maximum of 8 s to rate the valence of the emotion on a 7-point scale (“–3”: very unpleasant, “0”: neutral, “+3”: very pleasant) by pressing on two keypads held in their hands. Participants were unaware that their memory for the pictures would be subsequently probed. Between the trials, a fixation cross (3.75° × 3.75°) was displayed on a light background. The latter ensured a relative pupillo-constriction, allowing a better detection of pupillary dilatation related to stimulus presentation. Forty null events, consisting in the presentation of the fixation cross during 6 s, were randomly introduced between the other trials.

During the retest session, 40 previously presented pictures of each category (pleasant, unpleasant, and neutral) were chosen for each subject and presented as “old” items during the retest. Twenty other pictures of each category, which had not been presented during encoding, were used as new items. The 180 pictures were presented in random order. Each picture was displayed for 2 s (17° × 23°) and participant had a maximum of 8 s to choose one of three possible choices: “Remember,” “Know,” or “New”. A “Remember” response indicated that the recognition of the item was associated with retrieval of specific contextual details during encoding (for example, the rating of emotional valence). A “Know” response was associated with the feeling of having encoded the item but without being able to retrieve any further specific details. A “New” response was given when the participant thought the item had not been presented during encoding. Participants gave their responses on a three-button keypad which they held in their right hand. In a debriefing after the retest, we asked the subjects to justify their “Remember” and “Know” responses to ensure that they understood the instructions properly.

The three first pictures of each session were used for habituation and were not modeled in the design matrix. Again, 60 null events were randomly introduced between the other trials. At the end of the session, outside the scanner, subjects rated the 60 new pictures for emotional valence with the 7-point scale used during the encoding.

For the encoding session, subjects were scanned on the afternoon of day 1, between 3:30 and 8:30 p.m., to reduce interferences before the night. For the retest session, they were scanned on day 4 at the same time of day as for encoding session.

Subjects were split in two groups according to whether they were allowed to sleep (resting sleep, RS, 21 volunteers, 12 women and 9 men, mean age 22.6 ± 3.0 years) or were totally sleep deprived (TSD, 18 volunteers, 11 women and 7 men, mean age 21.9 ± 3.9 years) during the first post-encoding night. In the RS group, subjects went home after the encoding session and slept regularly as during the week before and during the three post-encoding nights. In the TSD group, the subjects stayed awake in the laboratory during the first post-encoding night (from 11.00 p.m. to 7.00 a.m.). During this night, the subjects remained under the constant supervision of experimenters. Light was kept under 30 lux [55]. Subjects were sitting on an armchair. We played board games during the whole night to keep them awake. Their physical activity was maintained as low as possible and followed a regular schedule. Every hour, subjects were allowed to stand up and to eat a small-standardized snack. During the following day, subjects were instructed to continue their usual activities. They slept at home during the two remaining nights. Participants were informed of their attribution to RS or TSD group only after the end of the encoding session.

### Data acquisition.

Data were acquired with a 3-T head-only magnetic resonance (MR) scanner (Allegra, Siemens) using a gradient echo-planar imaging (EPI) sequence [32 transverse slices with 30% gap, voxel size: 3.4 × 3.4 × 3.4 mm, repetition time (TR): 2130 ms, echo time (TE): 40 ms, flip angle (FA): 90°, field of view (FOV): 220 mm]. Between 420 and 480 functional volumes were acquired during encoding and between 490 and 570 functional volumes during retest. In all sessions, the first three volumes were discarded to account for magnetic saturation effects. A structural MR scan was acquired at the end of the experimental session (T1-weighted 3D MP-RAGE sequence, TR: 1960 ms, TE: 4.43 ms, TI: 1100 ms, FOV: 230 × 173 mm^2^, matrix size 256 × 192 × 176, voxel size: 0.9 × 0.9 × 0.9 mm). Stimuli were displayed on a screen positioned at the rear of the scanner, which the subject could comfortably see through a mirror mounted on the standard head coil.

During both acquisition sessions, eye movements and pupillary size were measured continuously using an infrared eye tracking system (LRO5000, Applied Science Laboratories, sampling rate: 60 Hz).

### fMRI data analysis.

fMRI data were analyzed using SPM2 (http://www.fil.ion.ucl.ac.uk) implemented in MATLAB 6.5 (Mathworks). Functional scans were realigned using iterative rigid body transformations that minimize the residual sum of square between the first and subsequent images. They were normalized to the MNI EPI template (2D spline, voxel size: 2 × 2 × 2 mm) and spatially smoothed with a Gaussian kernel with full-width at half maximum (FWHM) of 8 mm.

Data were processed using two-step analysis, taking into account the intraindividual and interindividual variance. For each subject, brain responses were modeled at each voxel, using a general linear model. During the retest session, nine trial types were modeled: old negative, positive, or neutral images correctly remembered (Neg-R, Pos-R, Neu-R); old negative, positive, or neutral images correctly identified as known (Neg-K, Pos-K, Neu-K); new negative, positive, or neutral images correctly identified as new (Neg-N, Pos-N, Neu-N). Additional trial types consisted of all forgotten images (misses), false alarms items, and the three first images. During encoding, six trials types were modeled: negative, positive, or neutral images subsequently correctly remembered (Neg-R, Pos-R, Neu-R); negative, positive, or neutral images subsequently correctly identified as known (Neg-K, Pos-K, Neu-K). Additional trial types consisted of subsequently forgotten images and the three first images. Each trial was categorized as neutral or emotional based on the individual subjective ratings. Negative images corresponded to scores “–3” and “–2,” neutral images correspond to “–1,”“0,” and “+1,” and positive images correspond to “+2” and “+3.” For each trial type, a given item was modeled as a delta function representing its onset. The ensuing vector was convolved with the canonical hemodynamic response function, and used as a regressor in the individual design matrix. Movement parameters estimated during realignment (translations in *x*, *y*, and *z* directions and rotations around *x-*, *y-*, and *z-*axes) and constant vector were also included in the matrix as a variable of no interest. High-pass filter was implemented using a cut off period of 128 s to remove the low-frequency drifts from the time series. Serial autocorrelations were estimated with a restricted maximum likelihood algorithm using an autoregressive model of order 1 (+ white noise). For the retest session, linear contrasts estimated the main effect of emotion (negative versus neutral or positive versus neutral) and memory (remember versus know). However, the contrasts of interest were essentially the emotion by memory interaction. [(Neg-R – Neu-R) – (Neg-K – Neu-K)] and [(Pos-R – Neu-R) – (Pos-K – Neu-K)]. The resulting set of voxel values constituted a map of *t* statistics [SPM(T)]. For the encoding session, the contrast of interest was the main effect of emotion (negative versus neutral). The individual summary statistical image were spatially smoothed with a Gaussian kernel with FWHM of 6 mm and used in a second-level analysis, corresponding to a random-effect analysis. The second-level analysis consisted in one-sample *t*-tests testing for the effect of interest separately for each group and in two-sample *t*-tests comparing the responses between the two groups (RS versus TSD and TSD versus RS). The latter contrasts corresponded to an emotion × memory × sleep status interaction. The resulting set of voxel values was thresholded at *p* < 0.001 (uncorrected). Statistical inferences were corrected for multiple comparisons using Gaussian random field theory at the voxel level in a small spherical volume (radius 10 mm) around a priori locations of structures of interest taken from the literature (Text S1).

A further multiregression analysis was conducted at the random-effect level to assessed whether the brain responses induced by successful recollection of emotional (negative respectively positive) versus neutral was linearly related to the global individually memory performance (number of correctly remembered images), and differentially so in sleep and sleep deprived subjects.

### Bayesian inferences and posterior probability maps.

In the random-effects analyses, we also computed posterior probability maps (PPMs) enabling conditional or Bayesian inferences about regionally specific effects [56]. PPMs and effect size were computed for the contrast of the interaction between emotion and memory in a given group (RS or TSD) to verify that seed areas of each group (respectively the hippocampus and the amygdala) are not activated in the other group.

### Psychophysiological interaction analyses.

Psychophysiological interaction (PPI) analyses were computed to test the hypothesis that functional connectivity between seed regions (the bilateral hippocampi and the amygdala, see Results) and the rest of the brain not only differed between emotional (negative respectively positive) and neutral recognized items but was also influenced by the sleep status during the first post-encoding night (RS or TSD). For each individual, the coordinates of the seed areas corresponded to the local maxima detected within 10 mm of the peak voxel of the random effect (RFX) analysis.

A new linear model was prepared for PPI analyses at the individual level, using three regressors. One regressor represented the emotional status of remembered pictures (Neg-R versus Neu-R) or (Pos-R versus Neu-R). The second regressor was the activity in the reference areas. The third regressor represented the interaction of interest between the first (psychological) and the second (physiological) regressors. To build this regressor, the underlying neuronal activity was first estimated by a parametric empirical Bayes formulation, combined with the psychological factor and subsequently convolved with the hemodynamic response function [57]. The model also included movement parameters. A significant psychophysiological interaction indicated a change in the regression coefficients between any reported brain area and the reference region, related the retrieval of emotional (rather than neutral) stimuli correctly remembered. Next, individual summary statistic images obtained at the first level (fixed effects) analysis were spatially smoothed (6-mm FWHM Gaussian kernel) and entered in a second-level (random effects) analysis using two sample *t*-tests to compare the functional connectivity between groups. Inferences were conducted as for the main effect analysis.

### Analysis of behavioral data.

Memory performances were calculated by the percentage of old items correctly identified as remembered, old items correctly identified as familiar and new items identified as old (false alarms). Items were split according to the subjective rating of emotion of each subject (negative = score “–3,” “–2;” neutral = score “–1,” “0,” and “+1;” and positive = score “+2,”“+3”). A repeated measure ANOVA with memory (R versus K) and emotion (Neg, Neu, Pos) as within-subjects factors and sleep group (RS versus TSD) as between-subjects factor was performed to test the effect of the group of sleep on memory and emotion separately, the effect of emotion on memory, and the interaction between emotion, memory, and sleep.

To test for a possible confound of a recognition bias due to emotion or sleep on memory performance, we performed an ANOVA on false alarms, with emotion as within-subject factor and sleep group as between-subjects factor. We calculated also the discrimination index (d') and the criterion (C) according to the procedure of Snodgrass and Corwin [35] for each group of sleep. *t*-test were performed to compare groups with this variables (d' and C).

### Analysis of pupillary size data.

During both encoding and retest sessions, mean pupillary size was estimated during the second following the beginning of the picture display. During this interval, the pupillary size was stable enough to assess the autonomic arousal expected to reflect primarily the emotional valence of the images. Trials contaminated by blinks were discarded. To reduce the intersubject variability, baseline pupillary size was estimated during the null events (fixation cross), averaged, and subtracted from the mean values. A repeated measure ANOVA with emotion (neg, neu, pos) and session (encoding and retest) as within-subjects factors tested the effects of emotion and session and their interaction. One subject was discarded from the analysis because pupillary data of the retest session were not usable. Negative emotion corresponded to scores “–3” and “–2”, neutral emotion correspond to “–1,”“0,” “+1,” and positive emotion correspond to “+2” and “+3”. Planned comparisons tested the differences between negative versus neutral and positive versus neutral pupillary size during encoding and retest.

### Analysis of sleep parameters.

Mean sleep duration and quality of sleep were assessed by questionnaires. We performed a repeated measure ANOVA separately for these two factors, with the number of hour or the subjective rate of quality for three nights (night before encoding, second night after encoding, and night before retest) as within-subject factors and sleep (RS versus TSD) as between-subject factor. Planned comparisons tested the difference between both groups for the three nights separately. The first night after the encoding was not included in the analysis because there were no data for the TSD group on this night.

Actigraphy data were analyzed for the four nights before encoding and between the encoding and the retest session. Sleep periods were estimated by a decrease in movements. For the deprivation night, the mean of movement's intensity was estimated from 11 p.m. to 7 a.m., corresponding to the period where subjects stayed awake at the lab. We performed a repeated measure ANOVA with mean of movements for four periods of night (night before encoding, first, second, and third nights after encoding) as within-subject factors and sleep (RS versus TSD) as between-subject factor. Planned comparison tested the difference between both groups for the three nights separately.

## Supporting Information

Figure S1 Effect of Emotion (A) Main effect of correct recognition (R+K) for stimuli of the three different emotional valences during retest. From left to right: effect of negative, positive, and neutral stimuli, respectively. A large occipito-hippocampal region and frontal areas were commonly activated by all stimuli. Response in the amygdala is only elicited by negative stimuli. Functional results are displayed on the mean structural MR image, normalized to the same stereotactic space (display at *p* < 0.001 uncorrected).
(B) Main effect of emotion during retest. From left to right: negative versus neutral, positive versus neutral, negative versus positive contrasts. The occipital and fusiform cortex, the lateral frontal cortex, and the amygdala are more activated for negative than neutral or positive items. Functional results are displayed on the mean structural MR image, normalized to the same stereotactic space (display at *p* < 0.001 uncorrected).
(20.5 MB TIF)

Figure S2 Effect of Memory Main effect of memory (R>K) for all subjects during retest. The medial prefrontal, the posterior cingulate cortex/precuneus, and both of the hippocampi show significant responses. Functional results are displayed on the mean structural MR image, normalized to the same stereotactic space (display at *p* < 0.001 uncorrected).
(8.9 MB TIF)

Table S1 Sleep Parameters (mean ± SD) (23 KB DOC)

Table S2 Number of Pictures in Each Valence Category and Mean of the Valence for Each Category on a 7-Point Scale (from “–3” for Negative Pictures to “+3” for Positive Pictures) (20 KB DOC)

Table S3 Main Effect of Correct Recognition (R+K) for Stimuli of the Three Different Emotional Valences during Retest, All Subjects (RS and TSD Group) All regions are significant after correction for multiple comparisons over the entire brain volume, except the amygdala for which the correction was conducted over a 10-mm sphere centered on published coordinates. Negative (resp. positive) coordinates on the *x*-axis indicate a left-sided (resp. right-sided) response. MNI, Montreal Neurological Institute. Inf, infinite
(34 KB DOC)

Table S4 Effect of Emotion on Successful Recognition (R+K) during Retest, All Subjects (RS and TSD Group) *P*_svc_: significance after correction for multiple comparisons over a small volume of interest (10-mm sphere), except in areas labeled by an asterisk (*), for which significance survives correction for multiple comparisons over the entire brain volume.
(33 KB DOC)

Table S5 Effect of Memory (R>K) during Retest, all Subjects (RS and TSD Group) All regions are significant after correction for multiple comparisons over the entire brain volume.
(21 KB DOC)

Table S6 Interaction between Emotion and Memory (Neg>Pos) × (R>K) during the Retest *P*_svc_: significance after correction for multiple comparisons over a small volume of interest (10-mm sphere).
(26 KB DOC)

Text S1 Description of Subject Selection and Reference Coordinates Used for Statistical Inferences (200 KB DOC)

## Abbreviations

ANOVA: analysis of variance
fMRI: functional MRI
PPI: psychophysiological interaction
REM: rapid eye movement
RS: resting sleep
TSD: total sleep deprivation
