# Supplementary material for: Sleep-Related Hippocampo-Cortical Interplay during Emotional Memory Recollection
Source: PLoS Biol. 2007 Oct 23;5(11):e282. doi: 10.1371/journal.pbio.0050282 (PMC2039770; doi:10.1371/journal.pbio.0050282)
Supplement: Supplementary file 1 [file pbio.0050282.sd001.doc]

## Supplemental material and method

***1. Subject selection***

Participants had no medical, psychiatric, traumatic or sleep disorders. Their scores on Beck depression scale[1], Beck anxiety scale [2] and Bermond-Vorst Alexithymia Questionnaire[3] were within normal range. Extreme morning and evening types, as assessed by the Horne-Ostberg Questionnaire[4], were not included. None complained of excessive daytime sleepiness as assessed by the Epworth Sleepiness Scale[5] and of sleep disturbances as determined by the Pittsburgh Sleep Quality Index Questionnaire[6]. All participants were right-handed as indicated by the Edinburgh Inventory[7]. One subject of the TSD group was discarded before the analysis because of anatomical brain abnormalities. Eighteen subjects were eventually included in the TSD group. The self-assessed questionnaires (for depression, anxiety, alexithymia, sleepiness, sleep quality and circadian rhythms) and age did not revealed any significant difference in both groups (p > 0.5).

2***. Reference coordinates used for statistical inferences***

# 2.1. Effect of emotion

###### Main effect of correct recognition for negative stimuli during retest

###### Amygdala (24 –9 –15)[8], (27,-3,-18)[9]

###### Effect of emotion (Neg>Neu) during retest

Amygdala (30,2,-26)[10], Orbitofrontal cortex (-3,48,-21)[11], Medial prefrontal cortex (16,56,19)[12], Inferior frontal cortex (56,28,10)[13], Thalamus (15,-22,-1)[14], Anterior temporal cortex (-46,12,-29)[14], (36,24,-36)[15], Posterior cingulate cortex (-8,-34,32)[14]

###### Effect of emotion (Pos>Neu) during retest

Medial prefrontal gyrus (-10,62,10)[13]

###### Effect of emotion (Neg>Pos) during retest

Amygdala (30,2,-26) [10], Middle frontal gyrus (16,47,42)[16], Inferior frontal gyrus (-48,30,10)[17] (-55,31,3) [18], Thalamus (15,-22,-1)[14], Superior temporal sulcus (63,-1,-12)[19]

Effect of emotion (Neg>Neu) during encoding

RS group: Amygdala (30,2,-26)[10], Fusiform gyrus (48,-45,-27)[20], Middle occipital gyrus (48,-78,0)[21] (-43,-75,-2)[22], Posterior cingulate gyrus (8,-54,46)[23], Middle cingulate gyrus (10,-4,32)[24], Medial prefrontal cortex (-2,54,14)[12], Orbitofrontal cortex (0,55,-14)[18], Superior temporal sulcus (32,10,-26)[15], Insula (48,-10,0)[18]

TSD group: Amygdala (30,2,-26)[10], Fusiform gyrus (48,-45,-27)[20] (-29,-57,-14)[22], Middle occipital gyrus (48,-78,0)[21] (-43,-75,-2)[22], Posterior cingulate cortex(-1,-51,22)[22], Medial prefrontal cortex (4,48,40)[12], Orbitofrontal cortex (0,55,-14)[18], Precentral (37,-5,50)[22], Inferior temporal gyrus (-58,-16,16)[24] (-46,-4,-18)[23], Insula (41,-3,8)[22]

2.2. ***Effect of memory***

### All subjects

All this regions are significant for all the volume and corrected for multiple comparisons. We performed no SVC.

RS group:

Medial prefrontal cortex (9,63,21)[25], Posterior hippocampus (36,-38,-6) [26]

2.3. ***Interaction between emotion and memory***

###### Interaction between emotion and memory (Neg>Neu) x (R>K) during retest

RS group: Medial prefrontal cortex (8,64,16)[13], Superior frontal gyrus (22,4,64)[24], Anterior cingulate cortex (-10,34,20)[14], Cuneus (-8,-78,24)[17], Superior temporal sulcus (-64,-44,-2)[13], Hippocampus (30,-21,-21)[25], Middle prefrontal gyrus (-36,9,49)[26], Middle cingulate cortex (-7,-21,38) [18], Intraparietal sulcus (27,-54,51)[26]

TSD group: Superior temporal sulcus (51,-66,21)[8], Amygdala (8,-6,-24)[27], Fusiform gyrus (-32,-36,-26)[23]

###### Interaction between emotion and memory (Neg>Pos) x (R>K) during retest

RS group: Medial prefrontal cortex (8,64,16)[13], Superior temporal sulcus (64,-42,18)[17] (-48,-15,-6)[9], Posterior cingulate cortex (4,-54,18)[17] (-7,-21,38)[18], Precuneus (12,-60,36)[26]

Interaction between emotion and memory (Pos>Neu)x(R>K) during retest

RS group: Superior temporal sulcus (38,6,-36)[13], Superior parietal gyrus (36,-66,48)[25]

TSD group: Precentral (52,-22,54)[31] (-40,-2,30)[31], Insula (-42,-3,-6)[9],

Interaction between emotion and memory (Pos>Neu)x(R>K) modulated by performance during retest

RS group : Medial prefrontal cortex (-3,60,21) [28] (-2,32,-10)[32], Superior frontal gyrus (-6,45,51)[28], Hippocampus (-33,-27,-15) [8], (30,-21,-21)[25], Superior temporal sulcus (-48,-63,27)[28], Insula (–46,-6,12)[25]

###### Interaction between emotion and memory (Neg>Neu) x (R>K) modulated by performances during retest

RS group: Medial prefrontal cortex (-20,64,12)[17], Hippocampus (30,-21,-21)[25] , Posterior cingulate cortex/ precuneus (4,-54,22)[17], (9,51,-9)[8], Anterior cingulate cortex (7,28,28)[18], Middle temporal sulcus (63,-39,-9) [28], Precuneus (-15,-60,21)[28]

TSD group: Amygdala (20,-4,-24)[29], Middle temporal cortex(–33,-3,-39)[28], Superior temporal sulcus (-41,2,-10)[30], Orbitofrontal cortex (-52,30,-10)[17]

###### PPI on the hippocampus of the contrast (Neg>Neu) x (R>K)

RS group: Medial prefrontal cortex (-20,64,12)[17], Superior temporal sulcus (56,-2,-26)[27] (51,-6,-18)[8], Inferior temporal gyrus (-33,-3,-39)[28], Thalamus (-4,-18,14)[31]

###### PPI on the amygdala of the contrast (Neg>Neu) x (R>K)

TSD group : Orbitofrontal cortex (-18,36,-18)[26], Striate cortex (-15,-84,15)[8], Extrastriate cortex (-33,-93,3)[8], Superior temporal gyrus (-55,-52,10)[33], Insula (-38,-32,20)[23]

###### PPI on the hippocampus of the contrast (Pos>Neu) x (R>K)

RS group: Middle prefrontal cortex (-36,48,9)[28], Amygdala (–12,4,-26)[34], Hippocampus (30,-21,-21)[25]

TSD group: Middle frontal gyrus (24,21,51)[8]

3***. References***

1. Steer RA, Ball R, Ranieri WF, Beck AT (1997) Further evidence for the construct validity of the Beck depression Inventory-II with psychiatric outpatients. Psychol Rep 80: 443-446.

2. Beck AT, Epstein N, Brown G, Steer RA (1988) An inventory for measuring clinical anxiety: psychometric properties. J Consult Clin Psychol 56: 893-897.

3. Bermond B, Vorst HC, Vingerhoets AJ, Gerritsen W (1999) The Amsterdam Alexithymia Scale: its psychometric values and correlations with other personality traits. Psychother Psychosom 68: 241-251.

4. Horne JA, Ostberg O (1976) A self-assessment questionnaire to determine morningness-eveningness in human circadian rhythms. Int J Chronobiol 4: 97-110.

5. Johns MW (1991) A new method for measuring daytime sleepiness: the Epworth sleepiness scale. Sleep 14: 540-545.

6. Buysse DJ, Reynolds CF, 3rd, Monk TH, Berman SR, Kupfer DJ (1989) The Pittsburgh Sleep Quality Index: a new instrument for psychiatric practice and research. Psychiatry Res 28: 193-213.

7. Oldfield RC (1971) The assessment and analysis of handedness: the Edinburgh inventory. Neuropsychologia 9: 97-113.

8. Fenker DB, Schott BH, Richardson-Klavehn A, Heinze HJ, Duzel E (2005) Recapitulating emotional context: activity of amygdala, hippocampus and fusiform cortex during recollection and familiarity. Eur J Neurosci 21: 1993-1999.

9. Smith AP, Henson RN, Rugg MD, Dolan RJ (2005) Modulation of retrieval processing reflects accuracy of emotional source memory. Learn Mem 12: 472-479.

10. Morris JS, Dolan RJ (2004) Dissociable amygdala and orbitofrontal responses during reversal fear conditioning. Neuroimage 22: 372-380.

11. Smith AP, Stephan KE, Rugg MD, Dolan RJ (2006) Task and content modulate amygdala-hippocampal connectivity in emotional retrieval. Neuron 49: 631-638.

12. Heinzel A, Bermpohl F, Niese R, Pfennig A, Pascual-Leone A, et al. (2005) How do we modulate our emotions? Parametric fMRI reveals cortical midline structures as regions specifically involved in the processing of emotional valences. Brain Res Cogn Brain Res 25: 348-358.

13. Smith AP, Henson RN, Dolan RJ, Rugg MD (2004) fMRI correlates of the episodic retrieval of emotional contexts. Neuroimage 22: 868-878.

14. Damasio AR, Grabowski TJ, Bechara A, Damasio H, Ponto LL, et al. (2000) Subcortical and cortical brain activity during the feeling of self-generated emotions. Nat Neurosci 3: 1049-1056.

15. Lane RD, Chua PM, Dolan RJ (1999) Common effects of emotional valence, arousal and attention on neural activation during visual processing of pictures. Neuropsychologia 37: 989-997.

16. Kuchinke L, Jacobs AM, Vo ML, Conrad M, Grubich C, et al. (2006) Modulation of prefrontal cortex activation by emotional words in recognition memory. Neuroreport 17: 1037-1041.

17. Maratos EJ, Dolan RJ, Morris JS, Henson RN, Rugg MD (2001) Neural activity associated with episodic memory for emotional context. Neuropsychologia 39: 910-920.

18. Maddock RJ, Garrett AS, Buonocore MH (2003) Posterior cingulate cortex activation by emotional words: fMRI evidence from a valence decision task. Hum Brain Mapp 18: 30-41.

19. Tabert MH, Borod JC, Tang CY, Lange G, Wei TC, et al. (2001) Differential amygdala activation during emotional decision and recognition memory tasks using unpleasant words: an fMRI study. Neuropsychologia 39: 556-573.

20. Erk S, Kiefer M, Grothe J, Wunderlich AP, Spitzer M, et al. (2003) Emotional context modulates subsequent memory effect. Neuroimage 18: 439-447.

21. Schienle A, Schafer A, Hermann A, Walter B, Stark R, et al. (2006) fMRI responses to pictures of mutilation and contamination. Neurosci Lett 393: 174-178.

22. Simpson JR, Ongur D, Akbudak E, Conturo TE, Ollinger JM, et al. (2000) The emotional modulation of cognitive processing: an fMRI study. J Cogn Neurosci 12 Suppl 2: 157-170.

23. Vuilleumier P, Armony JL, Driver J, Dolan RJ (2003) Distinct spatial frequency sensitivities for processing faces and emotional expressions. Nat Neurosci 6: 624-631.

24. Morris JS, Friston KJ, Buchel C, Frith CD, Young AW, et al. (1998) A neuromodulatory role for the human amygdala in processing emotional facial expressions. Brain 121 ( Pt 1): 47-57.

25. Yonelinas AP, Otten LJ, Shaw KN, Rugg MD (2005) Separating the brain regions involved in recollection and familiarity in recognition memory. J Neurosci 25: 3002-3008.

26. Erk S, Martin S, Walter H (2005) Emotional context during encoding of neutral items modulates brain activation not only during encoding but also during recognition. Neuroimage 26: 829-838.

27. Sterpenich V, D'Argembeau A, Desseilles M, Balteau E, Albouy G, et al. (2006) The locus ceruleus is involved in the successful retrieval of emotional memories in humans. J Neurosci 26: 7416-7423.

28. Henson RN, Hornberger M, Rugg MD (2005) Further dissociating the processes involved in recognition memory: an FMRI study. J Cogn Neurosci 17: 1058-1073.

29. Kilpatrick L, Cahill L (2003) Amygdala modulation of parahippocampal and frontal regions during emotionally influenced memory storage. Neuroimage 20: 2091-2099.

30. Kensinger EA, Schacter DL (2005) Retrieving accurate and distorted memories: neuroimaging evidence for effects of emotion. Neuroimage 27: 167-177.

31. Ranganath C, Johnson MK, D'Esposito M (2003) Prefrontal activity associated with working memory and episodic long-term memory. Neuropsychologia 41: 378-389.

32. Takashima A, Petersson KM, Rutters F, Tendolkar I, Jensen O, et al. (2006) Declarative memory consolidation in humans: a prospective functional magnetic resonance imaging study. Proc Natl Acad Sci U S A 103: 756-761.

33. Narumoto J, Okada T, Sadato N, Fukui K, Yonekura Y (2001) Attention to emotion modulates fMRI activity in human right superior temporal sulcus. Brain Res Cogn Brain Res 12: 225-231.

34. Dolan RJ, Lane R, Chua P, Fletcher P (2000) Dissociable temporal lobe activations during emotional episodic memory retrieval. Neuroimage 11: 203-209.
