# Supplementary material for: Sleep-Related Hippocampo-Cortical Interplay during Emotional Memory Recollection
Source: PLoS Biol. 2007 Oct 23;5(11):e282. doi: 10.1371/journal.pbio.0050282 (PMC2039770; doi:10.1371/journal.pbio.0050282)
Supplement: Supplementary file 2 [file pbio.0050282.st001.doc]

Table S1: Sleep parameters (mean ± SD).

|  | Group | **Night before encoding** | **Night 1, after encoding** | Night 2 | **Night 3, before retest** |
| --- | --- | --- | --- | --- | --- |
| **Hours of sleep** | ***RS*** | 7.8 ±1.4 | 8.2 ±1.2 | 8.0 ±1.3 | 8.1 ±1.2 |
|  | ***TSD*** | 8.0 ±1.3 | - | 9.9 ±1.6 * | 8.6 ±1.4 |
| **Quality of sleep** | ***RS*** | 6.9 ±1.6 | 7.2 ±1.5 | 6.7 ±1.9 | 7.1 ±1.1 |
|  | ***TSD*** | 6.9 ±1.6 | - | 7.4 ±1.4 | 7.1 ±0.8 |
| **Actigraphy (arbitrary units)** | ***RS*** | 19.3 ±9.9 | 22.5 ±14.4 | 20.4 ±11.3 | 20.2 ±9.2 |
|  | ***TSD*** | 17.7 ±15.6 | 265.5 ±75.1 * | 13.9 ±6.8 * | 15.9 ±7.9 |
