# Supplementary material for: Sleep-Related Hippocampo-Cortical Interplay during Emotional Memory Recollection
Source: PLoS Biol. 2007 Oct 23;5(11):e282. doi: 10.1371/journal.pbio.0050282 (PMC2039770; doi:10.1371/journal.pbio.0050282)
Supplement: Supplementary file 3 [file pbio.0050282.st002.doc]

Table S2: Number of pictures in each valence category and mean of the valence for each category on a 7-point scale (from “–3” for negative pictures to “+3” for positive pictures)

| Number of images | Negative | Neutral | **Positive** |
| --- | --- | --- | --- |
| **Sleep group** | 28.0 ± 6.3 | 65.1 ± 17.3 | 26.9 ± 13.1 |
| **Sleep deprived group** | 30.8 ± 10.4 | 58.2 ± 19.0 | 30.7 ± 11.4 |

| Valence of emotion | Negative | Neutral | **Positive** |
| --- | --- | --- | --- |
| **Sleep group** | -2.45 ± 0.15 | 0.14 ± 0.15 | 2.33 ± 0.19 |
| **Sleep deprived group** | -2.50 ± 0.23 | 0.09 ± 0.10 | 2.34 ± 0.22 |

An ANOVA computed on the valence of emotion measured during encoding (neg :”-3”,”-2”, neu: “-1”,”0”,”+1”, pos: “+2”,”+3”) shows a main effect of emotion for all subjects (F(2,76)= 5534, p<0.001). Post-hoc comparisons revealed that negative and positive emotions induce a significant difference in valence as compared to neutral (neg>neu: F=4171, p<0.001, pos<neu: F=3420, p<0.001).
