# Supplementary material for: Sleep-Related Hippocampo-Cortical Interplay during Emotional Memory Recollection
Source: PLoS Biol. 2007 Oct 23;5(11):e282. doi: 10.1371/journal.pbio.0050282 (PMC2039770; doi:10.1371/journal.pbio.0050282)
Supplement: Supplementary file 4 [file pbio.0050282.st003.doc]

Table S3: **Main effect of correct recognition (R+K) for stimuli of the 3 different emotional valences during retest**, all subjects (RS and TSD group)

All regions are significant after correction for multiple comparisons over the entire brain volume, except the amygdala for which the correction was conducted over a 10-mm sphere centered on published coordinates. Negative (resp. positive) coordinates on the x axis indicate a left-sided (resp. right-sided) response. **MNI** : Montreal Neurological Institute. **Inf** = infinite

|  | ***Effect of negative stimuli*** | | | ***Effect of positive stimuli*** | | | ***Effect of neutral stimuli*** | | |
| --- | --- | --- | --- | --- | --- | --- | --- | --- | --- |
| **Brain regions** | **MNI coordinates**  **(x,y,z:mm)** | **Z-score** | **P** | **MNI coordinates**  **(x,y,z:mm)** | **Z-score** | **P** | **MNI coordinates**  **(x,y,z:mm)** | **Z-score** | **P** |
| Amygdala | 24 –18 –14  18, -2, -24 | 5,27  4,28 | <0.001  0,001 |  |  |  |  |  |  |
| **Fusiform gyrus** | -32,-76,-16  36,-82,-14  32,-56,-16  -36,-52,-22 | Inf  Inf  Inf  Inf | <0.001  <0.001  <0.001  <0.001 | -24,-80,-14  -28,-74,-16  -38,-52,-22  32,-56,-14 | Inf  Inf  Inf  Inf | <0.001  <0.001  <0.001  <0.001 | -24,-80,-14  36,-82,-12  -36,-48,-24  32,-54,-14 | Inf  Inf  Inf  Inf | <0.001  <0.001  <0.001  <0.001 |
| **Thalamus** | -10,-18,6  12,-6,0 | Inf  Inf | <0.001  <0.001 | -10,-14,4  8,-12,0 | Inf  Inf | <0.001  <0.001 | -10,-18,2  14,14,0 | Inf  Inf | <0.001  <0.001 |
| **Hippocampus** | 22,-28,-4  -22,-28,-6 | Inf  Inf | <0.001  <0.001 | -18,-30,-6  22,-28,-4 | Inf  Inf | <0.001  <0.001 | -20,-28,-6  22,-28,-2 | Inf  Inf | <0.001  <0.001 |
| **Cuneus** | 4,-80,8 | Inf | <0.001 | 0,-84,2 | Inf | <0.001 | -8,-96,0 | Inf | <0.001 |
| **Medial prefrontal cortex** | -2,16,48 | Inf | <0.001 | -2,18,48 | Inf | <0.001 | -4,18,48 | Inf | <0.001 |
| **Dorsolateral prefrontal gyrus** | -52,8,38  42,8,32 | Inf  Inf | <0.001  <0.001 | -50,8,38  42,8,32 | Inf  Inf | <0.001  <0.001 | -52,8,38  42,8,32 | Inf  Inf | <0.001  <0.001 |
| **Orbitofrontal gyrus** | -40,28,-22  32,36,-18 | Inf  Inf | <0.001  <0.001 | -32,20,-8  34,24,-8 | Inf  Inf | <0.001  <0.001 | -34,20,-8  34,20,-6 | Inf  Inf | <0.001  <0.001 |
| **Parietal gyrus** | -28,-52,50  34,-54,56 | Inf  Inf | <0.001  <0.001 | -30,-52,50  28,-58,52 | Inf  Inf | <0.001  <0.001 | -30,-62,48  26,-58,50 | Inf  Inf | <0.001  <0.001 |
