# Supplementary material for: Sleep-Related Hippocampo-Cortical Interplay during Emotional Memory Recollection
Source: PLoS Biol. 2007 Oct 23;5(11):e282. doi: 10.1371/journal.pbio.0050282 (PMC2039770; doi:10.1371/journal.pbio.0050282)
Supplement: Supplementary file 5 [file pbio.0050282.st004.doc]

Table S4: Effect of **emotion on successful recognition (R+K) during retest**, all subjects (RS and TSD group)

**P_svc_** : significance after correction for multiple comparisons over a small volume of interest (10mm sphere), except in areas labeled by a ‘*’ for which significance survives correction for multiple comparisons over the entire brain volume.

|  | **Neg>Neu** |  |  | ***Pos>Neu*** |  |  | ***Neg>Pos*** |  |  |
| --- | --- | --- | --- | --- | --- | --- | --- | --- | --- |
| **Brain regions** | **MNI coordinates**  **(x,y,z:mm)** | **Z-score** | **P_svc_** | **MNI coordinates**  **(x,y,z:mm)** | **Z-score** | **P_svc_** | **MNI coordinates**  **(x,y,z:mm)** | **Z-score** | **P_svc_** |
| Amygdala | 34, -4, -30 | 3.55 | 0.014 |  |  |  | 28,-4,-28 | 3.82 | 0.007 |
| **Orbitofrontal cortex** | 2,50,-24 | 3.54 | 0.014 |  |  |  |  |  |  |
| **Medial prefrontal cortex** | 8,60,24 | 3.79 | 0.007 | -8,66,6 | 3.77 | 0.008 | 4,62,30 | 4.89 | 0.029* |
| **Inferior frontal cortex** | 52,36,8 | 4.09 | 0.002 |  |  |  | 48,34,4  -48,32,2 | 3.63  3.30 | 0.012  0.031 |
| **Fusiform gyrus** | 48,-44,-22  -42,-44,-22 | 5.07  4.93 | 0.012*  0.022* |  |  |  | 48,-46,-26  -42,-46,-24 | 5.99  5.33 | <0.001*  0.004* |
| **Middle occipital gyrus** | 48,-72,6  -52,-74,12 | 5.88  6.15 | <0.001*  <0.001* |  |  |  | 52,-66,14  48,-62,-6  -52,-74,12  -50,-74,0 | 5.38  5.76  5.69  5.66 | 0.003*  <0.001*  0.001*  0.001* |
| **Thalamus** | 8,-28,-2 | 3.38 | 0.023 |  |  |  | 18,-26,-8 | 3.75 | 0.008 |
| **Superior temporal gyrus** | 42,16,-34  -44,18,-36 | 3.3  3.41 | 0.029  0.021 |  |  |  | 58,-4,-22 | 3.83 | 0.006 |
| **Posterior cingulate cortex** | -6,-58,30 | 3.28 | 0.03 |  |  |  |  |  |  |
