# Supplementary material for: Sleep-Related Hippocampo-Cortical Interplay during Emotional Memory Recollection
Source: PLoS Biol. 2007 Oct 23;5(11):e282. doi: 10.1371/journal.pbio.0050282 (PMC2039770; doi:10.1371/journal.pbio.0050282)
Supplement: Supplementary file 6 [file pbio.0050282.st005.doc]

Table S5: Effect of **memory (R>K)** during retest, all subjects (RS and TSD group)

All regions are significant after correction for multiple comparisons over the entire brain volume.

| **Brain regions** | **MNI coordinates**  **(x,y,z:mm)** | **Z-score** | **P** |
| --- | --- | --- | --- |
| Medial prefrontal cortex | -12,54,12 | 7.14 | <0.001 |
|  | -6,46,-12 | 6.38 | <0.001 |
|  | -6,62,32 | 6.33 | <0.001 |
|  | -2,36,4 | 6.09 | <0.001 |
| **Dorsolateral prefrontal cortex** | -34,-14,46 | 4,84 | 0,028 |
| **Cingulate cortex/precuneus** | -8,-26,46 | 6,89 | <0.001 |
| **Posterior cingulate cortex** | -6,-58,22 | 6,38 | <0.001 |
| **Hippocampus** | -28,-24,-20 | 5.33 | 0.003 |
| **Inferior temporal gyrus** | -52,-2,-32  58,-6,-18 | 5,99  6,10 | <0.001  <0.001 |
| **Superior temporal sulcus** | 68,-28,18 | 6.09 | <0.001 |
| **Angular gyrus** | -48,-68,30 | 6,08 | <0.001 |
