# Supplementary material for: Sleep-Related Hippocampo-Cortical Interplay during Emotional Memory Recollection
Source: PLoS Biol. 2007 Oct 23;5(11):e282. doi: 10.1371/journal.pbio.0050282 (PMC2039770; doi:10.1371/journal.pbio.0050282)
Supplement: Supplementary file 7 [file pbio.0050282.st006.doc]

Table S6: Interaction between emotion and memory **(Neg>Pos) x (R>K)** during the retest

**P_svc_** : significance after correction for multiple comparisons over a small volume of interest (10mm sphere).

| **Brain regions** | **MNI coordinates**  **(x,y,z:mm)** | Z-score | **P_svc_** | **MNI coordinates**  **(x,y,z:mm)** | Z-score | P_svc_ |
| --- | --- | --- | --- | --- | --- | --- |
| **One sample t-test** | **RS group** |  |  | **TSD group** |  |  |
| **Medial prefrontal cortex** | 8,64,12 | 3.44 | 0.031 |  |  |  |
| **Superior temporal sulcus** | 64,-46,24 | 4.18 | 0.003 | / |  |  |
| **Two sample t-test** | **RS > TSD** |  |  | **TSD > RS** |  |  |
| **Medial prefrontal cortex** | 12,66,8 | 3.25 | 0.03 | / |  |  |
| **Superior temporal sulcus** | -56,-22,-10 | 3.47 | 0.019 |  |  |  |
| **Posterior cingulate cortex** | -12,-22,34  8,-52,22 | 3.85  3.43 | 0.006  0.021 |  |  |  |
| **Precuneus** | 6,-54,40 | 3.41 | 0.022 |  |  |  |
